# Effect of poly‐L‐lactic acid and polydioxanone biostimulators on type I and III collagen biosynthesis

**DOI:** 10.1111/srt.13681

**Published:** 2024-04-08

**Authors:** Rachel Thacyana Rorato Bernardo, Renata Cristina Gobbi de Oliveira, Karina Maria Salvatore de Freitas, José Ricardo de Albergaria‐Barbosa, Célia Marisa Rizzatti‐Barbosa

**Affiliations:** ^1^ Centro Universitário Ingá, Uninga Paraná Brazil

**Keywords:** biostimulators, collagen, polydioxanone, poly‐L‐Latic acid, rejuvenation, skin, subcision

## Abstract

**Objective:**

Safe, effective, and biocompatible minimally invasive procedures with the potential to stimulate collagen production have been made to recover dermal thickness and skin quality. The main of this animal model experiment was to observe the effect of poly‐L‐lactic acid (PLLA) and polydioxanone (PDO) biostimulators in collagen I and III after hypodermal injection.

**Methodology:**

Sixteen adult female rats (*Wistar*) were randomized into four groups and had dorsal treatment with: G1: hypodermic subcision (HS) only; G2: HS and PLLA hypodermic injection (HI), G3: HS and PDO HI; G4: Control, with no treatment.

**Results:**

In histochemical, it was observed hypodermal and dermal tissue in more organized thickness in G3 and in G4 when compared to G1 and G2. There was few difference in G1 compared to G4. The tissue of G2 showed irregularities in the arrangement of collagen fibers, less defined structure and lower distribution of type I collagen compared to the other groups. There is a greater tendency for the proportions of type III collagen among tissues treated with both biostimulators (G2 and G3). PLLA and PDO had relatively similar percentages of collagen when compared to G4. The amount of type I collagen was higher in tissues treated with subcision, while type III collagen was higher in tissues treated with both biostimulators.

**Conclusion:**

G3 showed better performance in collagen production, although small, when compared with G2.

## 1 INTRODUCTION

Cutaneous aging is characterized by intrinsic changes due to the deficiency of cell regeneration, as well as by changes in dermal level, mainly during senescence, where dermis assume relatively acellular and avascular nature.^1^, ^2^ The thinning of the dermal thickness is a striking and irrefutable characteristic of the natural process of chronological aging, evidenced by a decrease in the fibers of collagen, elastin, and fundamental substances, such as hyaluronic acid.^3^, ^4^ Continuous degradation of collagen is observed due to the presence of high levels of collagenases and, in this condition, there is no balance between collagen synthesis and degradation. Consequently, the disorganization of the remaining fibers is evident, apparently thinner, and more granular. These events lead to the replacement of type I collagen for type III collagen, found in younger and older individuals, respectively.^4^, ^5^

Fibroblasts play an important role in collagen synthesis and extracellular matrix organization participating in skin morphogenesis, angiogenesis, and healing. Through the discovery of the participation of gene expression modulators, such as transforming growth factor type β (TGF‐β), it was possible to recognize its action in the expression of several genes of the extracellular matrix, serving as an adjunct in the coding of collagen I, III, IV, and V from the fibroblast.^6^ As there is a decline of growth factors in chronological aging and understanding that these are released by macrophages, some studies have proposed the induction of growth factors to stimulate collagen production with the intradermal application of biostimulators, known to induce controlled tissue inflammatory response,^5^, ^7^ since the replacement of decreased facial volume is the ideal step to achieve facial rejuvenation.^8^, ^9^, ^10^

Therefore, minimally invasive procedures aimed at facial rejuvenation have brought great versatility when choosing and using collagen biostimulators, which treat fine lines and wrinkles, while correcting the loss of volume of the aged face from the induction of collagenesis, resulting in improved thickness, sagging and dermal quality^11^

Inducing collagenesis through the intradermal application of poly‐L‐lactic acid (PLLA) has wide validation in the literature, which confirms its performance in correcting the decrease in volume, forming structural points for repositioning the soft tissues of the face.^12^, ^13^, ^14^, ^15^

Polydioxanone (PDO) is used as an absorbable material in the form of PDO threads to induce the formation of collagen.^16^ Recently, a new presentation of PDO powder is being marketed with the same indication of collagenesis activation; however, more detailed research on its performance in activating collagenesis and increasing dermal thickness and quality is still lacking.

To date, no experiments have been found comparing the effectiveness of PLLA and PDO biostimulators on skin collagenesis. In this way, the present study aimed to compare the effects of intradermal injection of PDO and PLLA in an animal model on the production of type I and III collagen in animal model.

## 2 METHODOLOGY

The experiment was approved by the Ethics Committee on the Use of Animals of the Ingá University Center (# 99/2020).

### 2.1 Experimental design

**FIGURE 1:**
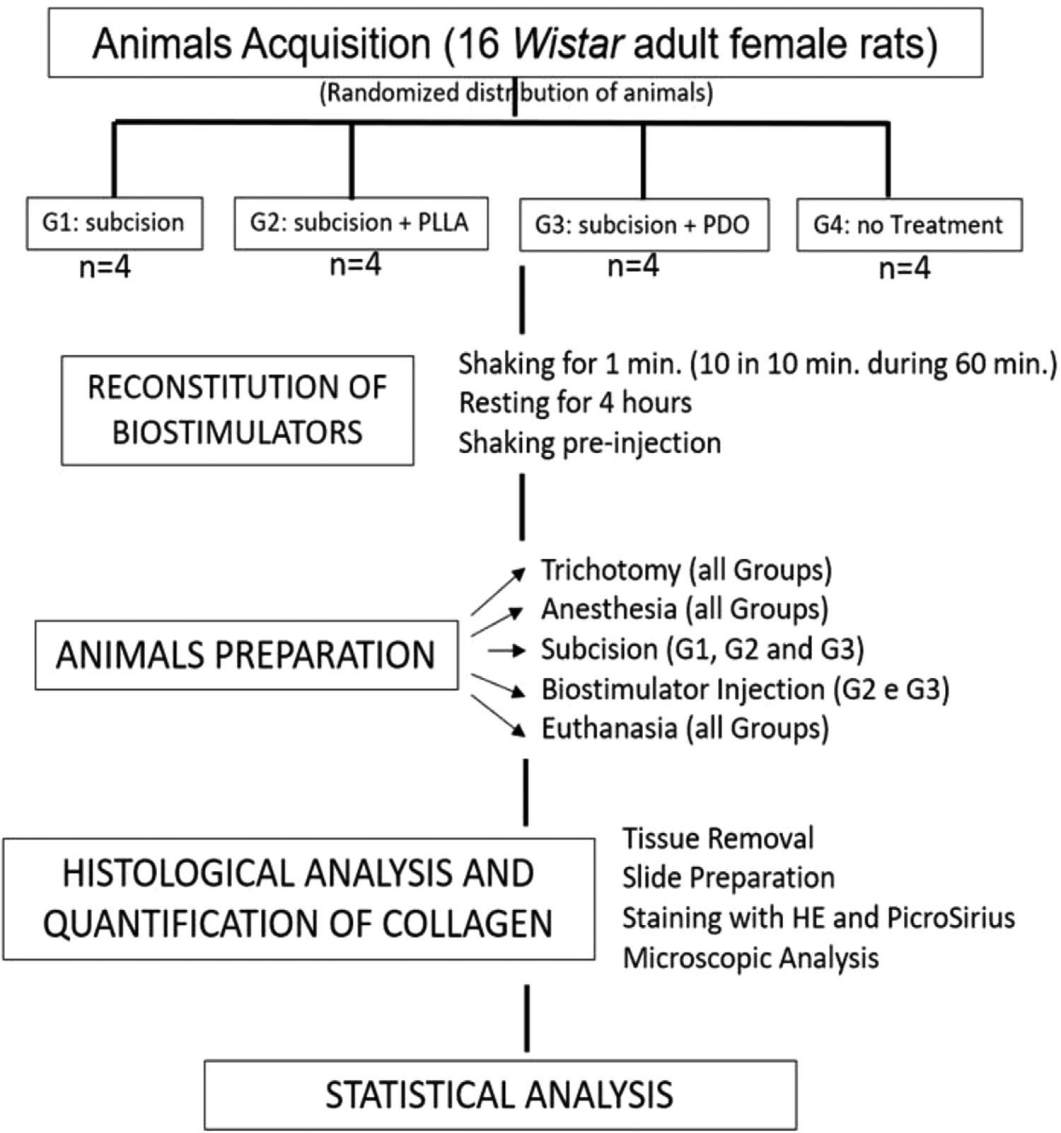
Flowchart of the experimental design of the study for induction of collagen formation in mice with subcision, subcision + PLLA, subcision + PDO, and no Treatment. G1, group 1; G2, group 2; G3, group3; G4, group 4; HE, hematoxylin and eosin; PDO, polydioxanone; PLLA, poly‐L‐lactic acid.

The flowchart summarizes the experimental steps of the study (Figure 1).

### 2.2 Biostimulators preparation

The reconstitution PLLA (Rennova Elleva, GANA R&D CO., Ltd, South Korea) and PDO (ULTRACOL, ULTRA V Co., Ltd, Korea) was performed with 14 mL of sterile distilled water under vigorous agitation for uniform powder mixing for 1 min every 10 min, until 1 h was completed. After the agitation time, the products were submitted to rest for 4 h. After the rest period, a new homogenization was performed, and the solution was used immediately. The remaining biostimulator solution was discarded.

### 2.3 Animals

Sixteen adult female rats (*Wistar*), weighing among 250–300 g, 4 months old, were randomized into four groups and had dorsal treatment with: G1 (*n* = 4): hypodermic subcision (HS) only; G2 (*n* = 4): HS and PLLA hypodermic injection (HI), G3 (*n* = 4): HS and PDO HI; G4 (*n* = 4): Control, with no treatment. The animals (2–3) were kept in cages (50 × 50 × 55 cm), in a closed environment, with cycle 12 h light/12 h dark, under 22–24°C, and humidity among 45%–65%. They received balanced commercial feed (Nuvital Nutrients Ltda) and water availability ad libitum, with standard sediment 53‐3, produced according to ISO 9001 (2008).

**FIGURE 2:**
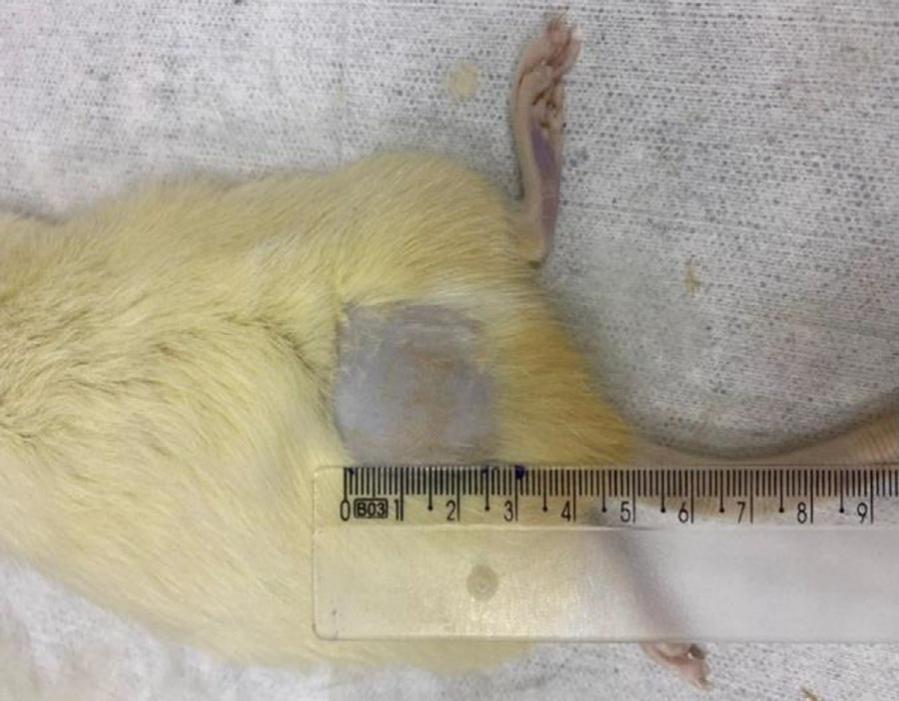
3 × 3 cm area demarcation in the posterior dorsal region for the treatment.

The treatments were applied to the posterior dorsal region. The demarcation of the area to be treated (3 × 3 cm) was performed by tattooing Henna (Figure 2). Within this delimitation, a previous trichotomy was performed and then it was done general anesthesia of the animal (11.6% ketamine hydrochloride: Dopalen – 50 mg/mL; and xylazine hydrochloride: Rompun – 2 g/100 mL) at 0.3 mL/100 g of body mass in a ratio of 1:1 intraperitoneal PI.

### 2.4 Treatment

In the animals of G1, only subcision was performed in the hypodermic plane with a 22G cannula (Rennova); in groups G2 and G3, 0.5 mL of biostimulator (PLLA and PDO, respectively) was injected after hypodermic subcision. The animals of G4 did not undergo any intervention.

The animals were kept in laboratory conditions for 30 days until new anesthesia for tissue collection.

### 2.5 Euthanasia and material collection

**FIGURE 3:**
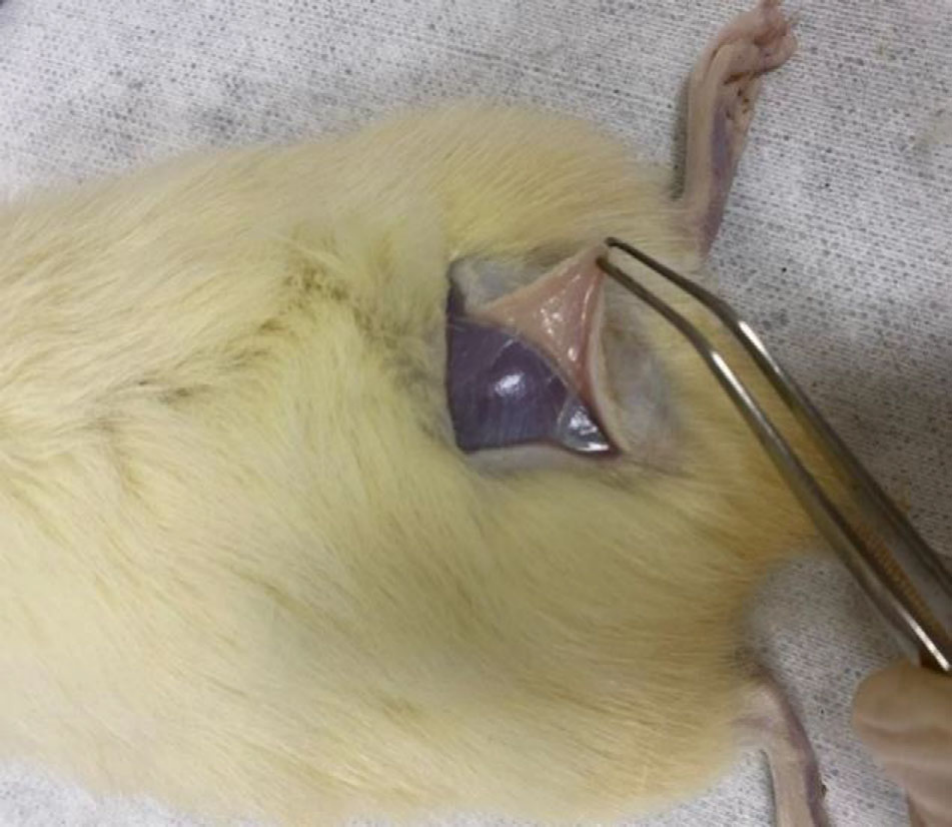
Tissue excision 30 days after experiment.

Euthanasia was conducted under general anesthesia by administering ketamine hydrochloride 50 mg and xylazine 10 mg intraperitoneally, followed by exsanguination by cardiac puncture. Soon after, 3 × 3 cm of tissue, including hypodermic plane, was removed for histological evaluation (Figure 3).

### 2.6 Histological slides

Histological analysis was performed 30 days after subcision and injection of biostimulators. The tissue fragments were fixed in 10% buffered formaldehyde, dehydrated in solutions of increasing concentrations of alcohols (70%, 80%, 90%, and 100%), diaphanized in xylol and soaked in paraffin. The histological sections, with a thickness of 5 μm, were obtained in a microtome (American Optical, model 820) and stained by the hematoxylin–eosin (HE) technique, for evaluation of the general morphology, and Picrosirius Red for the detection of type I and III collagens.^17^

For staining in Picrosirius, the slides were dewaxed in 100% xylene, washed with 100% ethanol and in water. Then, the slides were submerged in 0.1% Sirius Red F3BA (BDH Laboratory Supplies, Poole, UK) in saturated picric acid for 1 h at room temperature. Washing was performed to remove excess dye and immersed in 0.1 N HCl for 2 min followed by another wash in water. Finally, the slides were dehydrated with ethanol and xylene and dried at room temperature until microscopic reading.^18^

### 2.7 Microscopy and image capture

The histological slides were analyzed under an optical microscope (Nikon Eclipse, Shimjuku, Japan), coupled to a high‐resolution camera (Nikon Ds‐Fi1C, Shimjuku, Japan).

The digital images were captured in a specific program (NIS‐Elements version 4.0, Prague, Czech Republic). To visualize collagen, the histological sections were visualized under polarized light, in the 40X objective, where the stained collagen fibers exhibited coloration from bright green to red.^18^

### 2.8 Collagen quantification

The images obtained from the histological sections, stained with Picrosirius‐Red, were analyzed with the software Image‐Pro Plus (version 4.5 – Media Cybernetics). We evaluated 10 images per animal/group. In each image, the area occupied by type I collagen (red) and type III collagen (green) was determined separately. The results were expressed as the area occupied by each type of collagen (in percentage) in relation to the total area analyzed.^18^

### 2.9 Statistical analysis

Data normality was performed using the Kolmogorov–Smirnov test. Intergroup comparison was performed with the analysis of variance (ANOVA) test to a selection criterion and Tukey's test. Statistical analysis was performed using the Statistic 12.0 software (Statsoft, Tulsa, Okla, USA), and the data were considered significant for *p* < 0.05.

## 3 RESULTS

**FIGURE 4:**
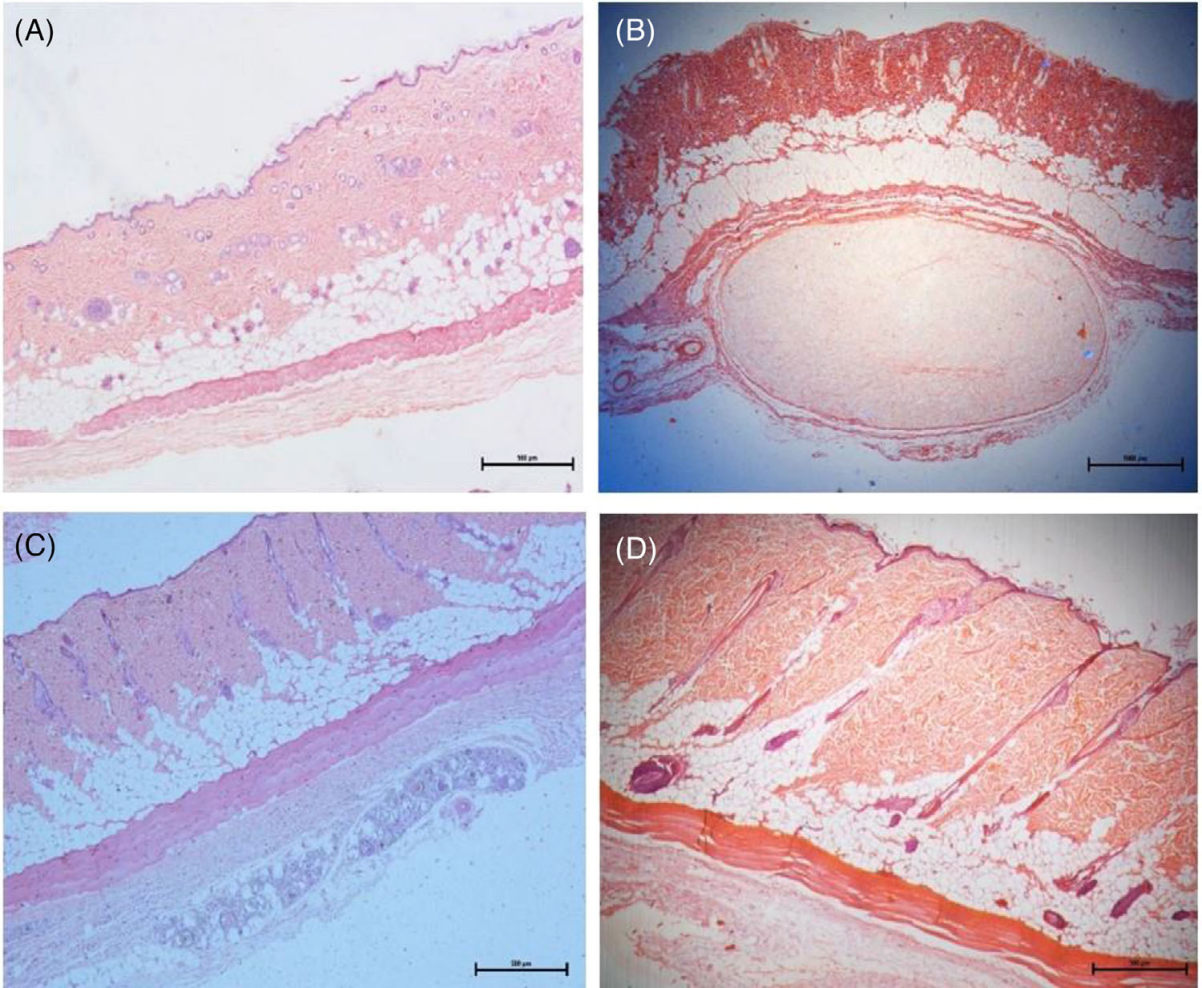
Tissue sections of the posterior dorsal region after 30 days of subcision, application of biostimulators and control group in HE stains. (A) Subcision with 22G Cannula (G1); (B) Subcision and treatment with 0.5 mL of PLLA in a single session (G2); (C) Subcision and treatment with 0.5 mL of PDO in a single session (G3); (D) Control with no treatment (G4). HE, hematoxylin–eosin; PDO, polydioxanone; PLLA, poly‐L‐lactic acid.

The histological sections after 30 days of treatment with only subcision (G1), biostimulators (G2–G3), and the control group (G4) can be seen in Figure 4.

The HE stains showed that the tissue presents a more organized thickness in the treatment performed with the PDO biostimulator (G3) (Figure 4C) and in the control group (G4) (Figure 4D), when compared to the subcision (G1) (Figure 4A) and PLLA (G2) (Figure 4B) groups.

In the tissue of the experimental group with PDO (G3) (Figure 4C), it is possible to verify well‐organized and compact collagen bundles. In contrast, tissue treated only with subcision (G1) (Figure 4A) and PLLA (G2) (Figure 4B) have less organized and compact collagen networks.

**FIGURE 5:**
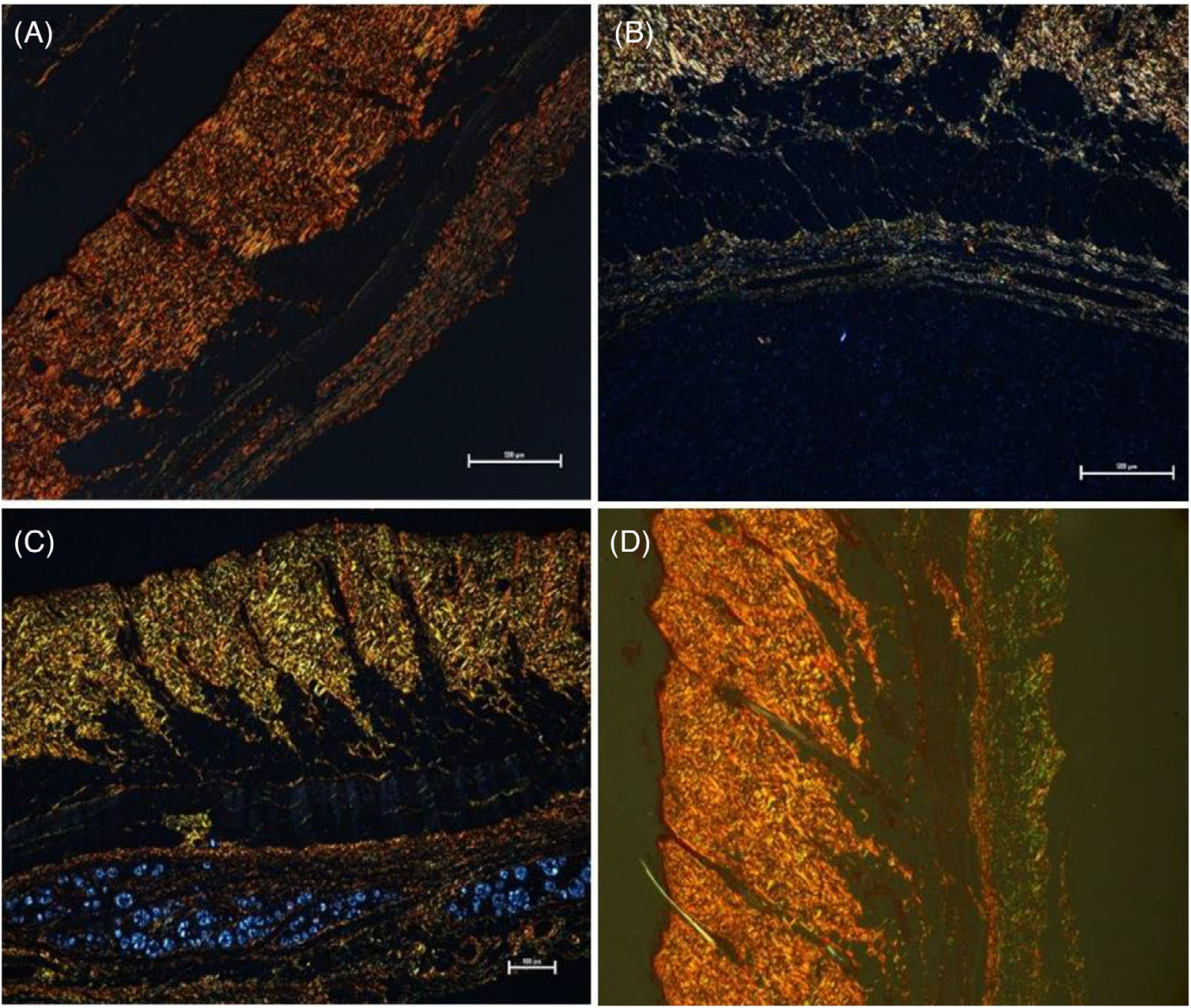
Tissue sections of the posterior dorsal region of mice after 30 days of subcision, application of biostimulators and control group in Picrosirius staining and visualized under polarized light. (A) Subcision with 22G Cannula (G1); (B) treatment with 0.5 mL of PLLA in a single session (G2); (C) treatment with 0.5 mL of PDO in a single session (G3); (D) control (G4). PDO, polydioxanone; PLLA, poly‐L‐lactic acid.

Under polarized light, it was possible to verify with greater clarity the collagen fibers of the analyzed tissues. As observed, the tissue treated only with subcision using a 22G cannula (G1) (Figure 5A) presented thick fibers that were predominantly yellow and red, indicating the presence of type I collagen.

The green fibers were evidenced in greater intensity in the tissues treated with biostimulators (Figure 5B, C: PLLA – G2; and PDO – G3, respectively), indicating the presence of type III collagen. It was also possible to notice that greater distribution of green fibers (indicative of type III collagen) was located in the tissues treated with PDO (G3) (Figure 5C). In addition, in Figure 5C it is possible to visualize the PDO microparticles in blue spherical format, indicating the induction of collagen formation in the region.

**FIGURE 6:**
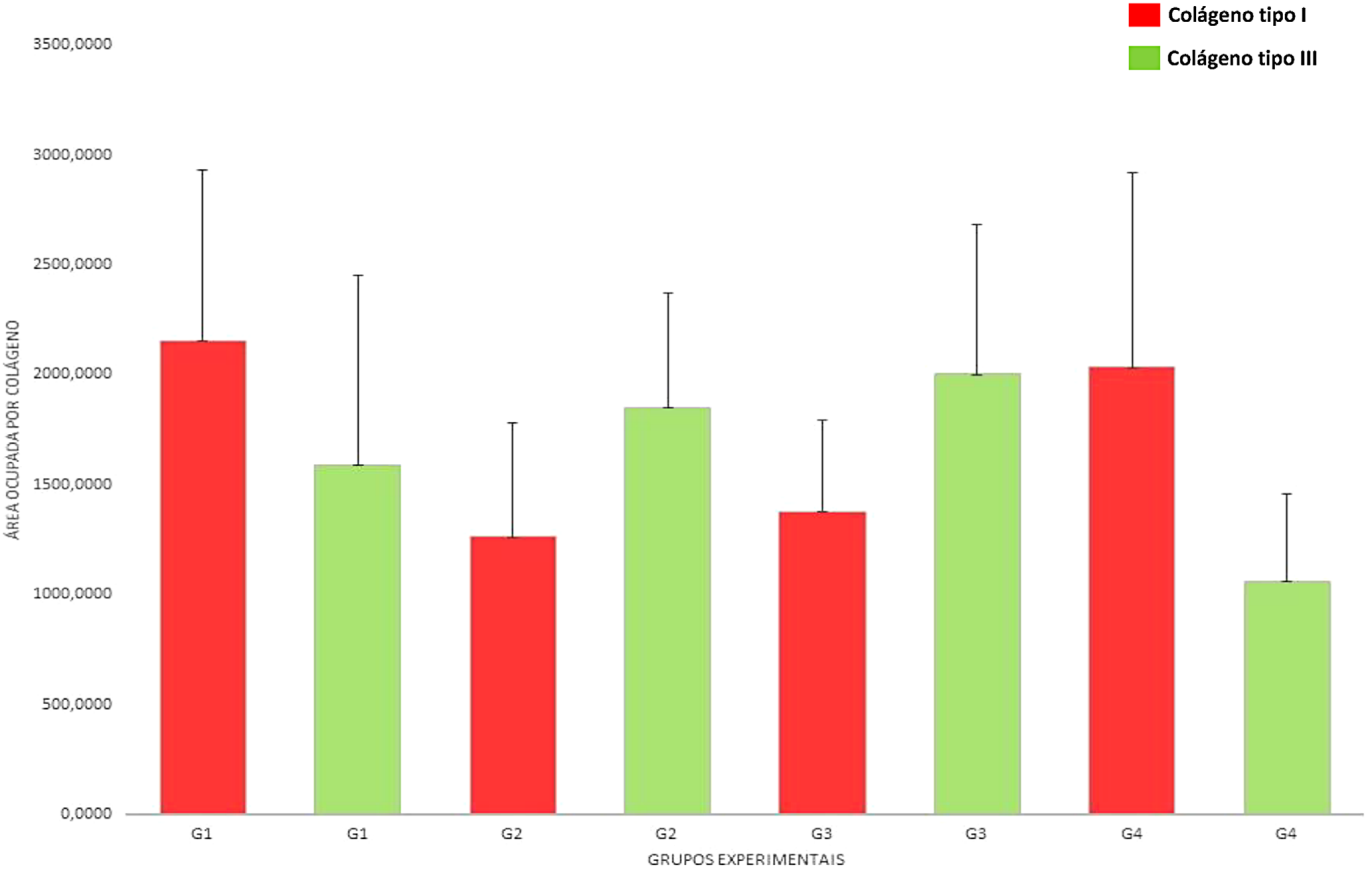
Proportion of red (type I collagen) green (type III collagen) staining in tissue sections of the posterior dorsal region of mice after 30 days of subcision, application of biostimulators and control group. G1: Subcision with 22G Cannula; G2: treatment with 0.5 mL of PLLA in a single session; G3: treatment with 0.5 mL of PDO in a single session; G4: control. The results are presented as average and standard deviation. PDO, polydioxanone; PLLA, poly‐L‐lactic acid.

The distribution pattern of collagen fibers for this study showed a slight difference in collagen stimulation after subcision with a 22G cannula (G1) (Figures 5A and 6), since the tissue kept its tissue architecture practically intact compared to the control group (G4) (Figures 5D and 6). On the other hand, the tissue treated with PLLA (G2) showed irregularities in the arrangement of collagen fibers, revealing a poorly defined structure and lower distribution of red color (type I collagen) compared to the other groups (Figure 5B).

All groups showed the presence of substantial green coloration (Figure 5A–D), which was confirmed after determining the proportions of green to red protein. However, the control group (G4) presented a lower percentage of type III collagen, green color (1062.90 ± 396) (Figure 6).

There is a greater tendency for the green proportions (type III collagen) among tissues treated with PLLA (G2) and PDO (G3) biostimulators (Figure 6), and there seems to be a relationship between biostimulator and higher biosynthesis of type III collagen in the recent collagen synthesis in this study.

The experimental groups (PLLA – G2; PDO – G3) presented relatively similar percentages of collagen when compared with the control group (G4), which were 23.33%, 25.35%, and 23.22%, respectively. Notably, G1 (subcision) showed a slight increase in the percentage of total collagen (28.09%), corresponding to a difference of 4.13% ± 0.9% (*p* = 0.000).

In the 30‐day period, type I collagen was higher with subcision and in the control group versus PLLA (G2) and PDO (G3) (*p* = 0.000). In the same period, the staining for type III collagen was higher with PDO, while the PLLA showed a similar type III collagen area (*p* = 0.000). Collagen values differ from each other when statistically compared between the different types of treatment and control after 30 days of intervention (Table 1).

**TABLE 1:** Intergroup comparison for collagen production (ANOVA to a selection criterion and Tukey's test).

Variables	G1	G2	G3	G4	*p*
	Average (SD)	Average (SD)	Average (SD)	Average (SD)	
SUM type I collagen	4558.03 (2016.03) A	2666.22 (1458.48) B	2909.65 (1317.84) B	4296.82 (2052.12) A	0.000^*^
Area type I collagen	2157.90 (954.44) A	1262.26 (690,48) B	1377.50 (623.90) B	2034.23 (971.53) A	0.000^*^
SUM type III collagen	2899.51 (2173.98) A	4060.95 (1259.94) B	4368.54 (1887.41) B	2245.13 (1113.62) A	0.000^*^
Area type III collagen	1590.24 (1071.27) A	1849.88 (705.94) AB	2068.19 (893.55) B	1062.90 (527.22) C	0.000^*^

Different letters on the same line indicate the presence of a statistically significant difference between the group indicated by the Tukey's test. ANOVA, analysis of variance; SD, standard deviation. ^*^Statistically significant for *p* < 0.05.

## 4 DISCUSSION

To the best of our knowledge, this study is an unprecedented research. It was demonstrated that the percentage of type I collagen is similar between the control group and the tissues treated with subcision using a 22G cannula, reflecting a significant difference in the percentage of type III collagen, 1060.90 and 1590.24 (*p* = 0.000), respectively.

Despite these findings, the quantification of collagen for subcision did not promote an expressive formation of collagen fibers when compared with the control group. Therefore, in the eyes of the evidence, little difference in skin quality will be achieved with subcision. Therefore, caution should be exercised when opting for the subcision technique with a single treatment modality, as suggested by other studies using this technique for the treatment of wrinkles and depressed scars.^19^

The small difference in collagen percentages between the experimental groups may be related to the analysis time, since the collagen stimulus is time dependent. That said, the results of the collagen stimulus may not be visualized for weeks, and it is necessary to wait for the results individually, because the response and the degree of improvement of the skin after the biostimulus will depend intrinsically on the characteristics of each patient, age, sex, and quality of the skin.^7^, ^20^

From this perspective, it is important that professionals qualified in injectable procedures on facial esthetic have this knowledge so as not to reproduce overcorrections with additional treatments early. In addition, when performing the treatment with collagen biostimulators, the patient should be advised that the result will be achieved more than 30 days after the injection of the biostimulator.^21^

As tissues mature, new collagen fibers are synthesized, increasing the percentage of total collagen. In addition, the green‐colored collagen fibers become thicker, replacing them with red‐colored fibers, thus increasing the areas of occupation of type I collagen.^18^

This mechanism may justify the trends in green‐to‐red protein ratios in our experimental trials (Figure 6). Possibly, the largest proportion of green‐stained proteins of groups G2 (PLLA) and G3 (PDO) are newly synthesized collagen fibers induced by biostimulators. Since the effect of biostimulators are late and gradual, being perceived by the dermal thickening promoted through collagenesis, forming type I collagen and in a smaller amount, type III collagen.^7^

The inflammatory reaction after the injection of collagen biostimulators initiates the deposition of type III collagen fibers around the microspheres of the material, followed by a fibroblastic tissue response and peripheral type I collagen deposition. This mechanism continues for weeks until there is remodeling of type III collagen with predominance of type I collagen in the newly formed tissue, resulting in improved dermal thickness and quality.^22^, ^23^

Studies on collagen synthesis from the injection of PLLA into the dermis of Wistar rats^24^ revealed that the biostimulator was able to stimulate the production of high content of type I collagen compared to the control group. This efficient behavior justifies its use to attenuate imperfections by stimulating collagen production in the skin.^24^

However, in this study, the area occupied by type I collagen for the same biostimulator was smaller than that of the control group. Once again, we reinforce the hypothesis that perhaps the experiment time was insufficient to determine the total potential of the biostimulator. Therefore, it is suggested that further in vivo studies with animal models be developed to test this hypothesis.

In Brazil, PDO is marketed in the form of smooth threads to stimulate collagen production and as spiculated wires promoting *liting* and facial rejuvenation. Smooth PDO wires act as a foreign body, stimulating the formation of subcutaneous fibrous tissue, which results in improved skin tension.^25^ Unlike smooth wires, the effect of spiculated wires is immediate, since it acts as a vertical vector for repositioning the soft tissues of the face.^26^ On the other hand, little is known about biocompatibility, safety, and efficacy of PDO powder. For this reason, this material was investigated in this study.

An investigation on the biostimulatory effect of the recently developed PDO^27^ revealed that PDO microspheres demonstrate a similar effect regarding collagenesis and inflammatory response when compared with PLLA and polycaprolactone (PLC). In addition, PDO also showed better degradability and significant decrease in skin roughness for the study. In this sense, these results, although incipient, open new avenues for the development of randomized clinical studies in humans to confirm in the future the safety and efficacy of the recently developed PDO powder‐based biostimulator, aiming at its use in clinical practice as a biostimulator of elastic and collagen fibers.

The technique of subcision and injection of biostimulators in the intradermal plane may have been one of the reasons for the equivalent amounts of collagen between the experimental groups. Histological studies also developed in animal models comparing collagen production after intradermal and subdermal injection of biostimulators revealed more significant amounts of collagen by the intradermal injection technique.^12^ Thus, these results serve as a basis for professionals to apply biostimulator intradermally to achieve better results, durability, and patient satisfaction.

Finally, the limitations of this study can be presented by the small number of animals used in the experiments and by the type of study model (in animals). On the other hand, the data show that the animal tissues treated with biostimulators revealed a greater presence of type III collagen, which was later replaced by type I collagen.^28^

In this sense, further randomized clinical studies are needed to determine whether this process of collagenesis occurs in humans after the injection of the biostimulators investigated, and whether these bring real benefits to esthetic treatments of collagen stimulation, improving dermal quality, in addition to determining the level of safety and efficacy of the biostimulators PLLA and PDO powder.

## 5 CONCLUSION

The performance of the biostimulators revealed that the PDO resulted in a more active collagenesis, stimulating greater production of collagen types I and III. The results of this study suggest that in the first month after treatment complete remodeling may not occur for the formation of new collagen fibers, which may have influenced the results regarding the type and amount of newly formed collagen when comparing the four experimental groups. Therefore, further studies are needed to determine the total performance of the biostimulators investigated in a longer period.

## Data Availability

The data that support the findings of this study are openly available in CV LATTES at http://lattes.cnpq.br/2135059544500650.
